# Impact of (long) COVID on athletes’ performance: a prospective study in elite football players

**DOI:** 10.1080/07853890.2023.2198776

**Published:** 2023-04-26

**Authors:** E. Wezenbeek, S. Denolf, J. G. Bourgois, R. M. Philippaerts, B. De Winne, T. M. Willems, E. Witvrouw, S. Verstockt, J. Schuermans

**Affiliations:** a Department of Rehabilitation Sciences, Ghent University, Ghent, Belgium; b Department of Movement and Sports Sciences, Faculty of Medicine and Health Sciences, Ghent University, Ghent, Belgium; c Department of Performance and Sports Sciences, KAA Ghent – UGent, Ghent, Belgium; d Royal Standard de Liège, Liège, Belgium; e Zulte Waregem, Waregem, Belgium; f Department of Electronics and Information Systems, Ghent University, Ghent, Belgium

**Keywords:** Aerobic endurance capacity, elite athletes, longitudinal cohort study, physical performance, SARS-CoV-2 infection, Yo-Yo test

## Abstract

**Objectives:**

To investigate possible persistent performance deficits after severe acute respiratory syndrome coronavirus 2 (SARS-CoV-2) infection in elite athletes.

**Methods:**

A prospective cohort study in three Belgian professional male football teams was performed during the 2020 − 2021 season. Participants were submitted to *strength, jump,* and sprint tests and an aerobic performance test (the Yo-Yo Intermittent Recovery test (YYIR)). These tests were repeated at fixed time intervals throughout the season. Assessment of SARS-CoV-2 infection was performed by a polymerase chain reaction (PCR) test before each official game.

**Results:**

Of the 84 included participants, 22 were infected with SARS-CoV-2 during follow-up. At the first testing after infection (52.0 ± 11.2 days after positive PCR testing) significantly higher percentages of maximal heart rate (%HRmax) were seen – within the isolated group of infected players- during (*p* = .006) and after the YYIR (2 min after, *p* = .013), compared to pre-infection data. This increase in %HRmax was resolved at the second YYIR testing after infection (127.6 ± 33.1 days after positive PCR testing). Additionally, when comparing the first test after infection in formerly infected to non-infected athletes, significantly higher %HRmax were found during (*p* < .001) and after the YYIR test (*p* < .001),No significant deficits were found for the jump, muscular strength or sprint tests.

**Conclusion:**

Aerobic performance seems compromised even weeks after infection. Simultaneously, anaerobic performance seemed to be spared. Because of the potential detrimental effects on the immune system, caution might be advised with high-intensity exposure until aerobic performance is restored.

**KEY MESSAGES**
1. Elite football players’ aerobic performance seems to be affected for weeks after they return to sports after a SARS-CoV-2 infection.
2. Similarly, anaerobic performance tests showed no discernible changes between both before and after SARS-CoV-2 infections.
3. Regular YYIR testing is recommended to monitor aerobic performance after SARS-CoV-2 infection.

## Introduction

Severe acute respiratory syndrome coronavirus 2 (SARS-CoV-2) is the coronavirus that causes ‘coronavirus disease 2019’ (COVID-19) [1]*.* COVID-19-related lockdowns led to marked reductions in athletic training specificity, intensity, frequency and duration but even to date, COVID-19 continues to worry athletes [2]*.* Although*,* athletes infected with severe acute respiratory syndrome coronavirus 2 (SARS-CoV-2) experience acute symptoms which are mostly mild or absent [3], emerging evidence suggests that a considerable proportion of athletes (3.8%–17.0%) may experience persistent symptoms [3]. This long COVID-19 syndrome (LC19), also called post-COVID-19 syndrome, or post-acute sequelae of infection by SARS-CoV-2, has been described previously, with approximately 1 in 10 individuals showing symptoms lasting ≥28 days [4] and a similar prevalence was reported in a cohort of athletes [3,5]. To date, the evidence and guidance regarding the rehabilitation and return to sport of this athlete population is rather limited [6,7]. A better understanding of LC19 in athletes is essential to inform safe measures and return to play (RTP) protocols, but currently the mid-to-long-term (i.e. weeks to months) impact of LC19 on athletes’ health and performance remains to be investigated [3].

The percentage of athletes not fully recovered from COVID-19 is significantly higher than for other acute respiratory diseases [3]. According to the review of Lemes et al. [3], professional athletes resume their sporting activities within 5–10 days after an asymptomatic or mild infection, which can be challenging for those who experience any symptoms upon RTP [1]. The most commonly reported long lasting symptoms in athletes are anosmia, dysgeusia and respiratory features as a persistent cough and lasting fatigue [3]. Therefore, sufficient rest and gradual RTP after infection in order to prevent cardiopulmonary complications has been suggested previously [7]. Also, a comprehensive clinical evaluation primarily focussed on cardiopulmonary assessments (i.e. blood analyses, electrocardiogram and spirometry) has been proposed for RTP clearance [8]. The study of Moulson et al. [9], found a remarkable high prevalence of abnormalities detected on spirometry in young athletes following COVID-19 [7]. Unfortunately, no pre-COVID-19 spirometry was available in this study, highlighting the need for prospective studies and regular athlete monitoring.

Since a SARS-CoV-2 infection might cause mild disease at onset but can develop into persisting (pulmonary) symptoms, possibly compromising athletes health and performance, adequate monitoring of the athletes seems imperative [3]. Because of the pathological effects of COVID-19 and associated quarantine, the hypotheses of this study was that regular physical testing of the athletes could identify (short and long term) decrements in performance related to infection, potentially affecting player’s health, injury risk and match availability*.* Therefore, the aim of this prospective cohort study was to investigate the role of regular field testing to identify possible persistent deficits in an athletic population after SARS-CoV-2 infection.

## Materials and methods

### Study design

**Figure 1.**
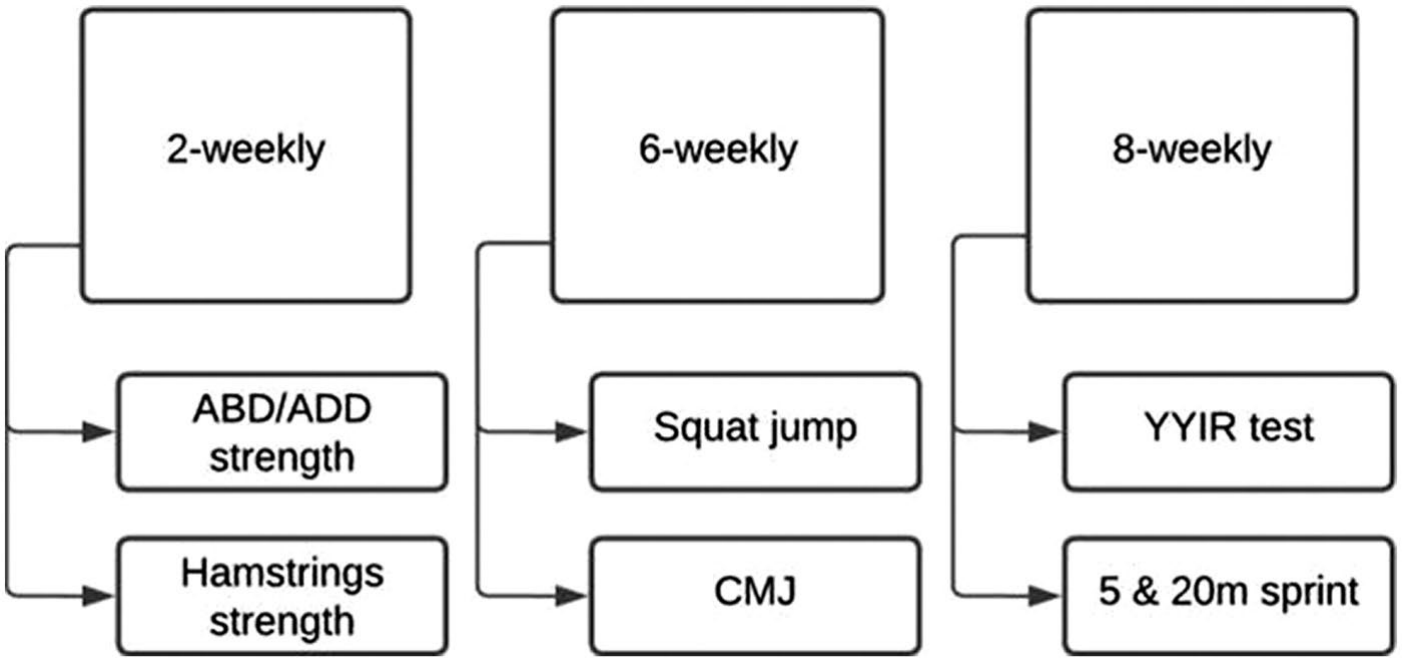
Overview monitoring physical performance tests. ABD: abduction; ADD: adduction; CMJ: countermovement jump; YYIR: Yo-Yo Intermittent Recovery Test; m: metre.

A prospective cohort study in Belgian professional male football players was performed during the 2020–2021 season (July 2020–May 2021). All players were submitted to an extensive screening battery that was conducted preseason and at regular time intervals during the season (Figure 1). In addition, all players were tested weekly for SARS-CoV-2 infection. General player information such as age, body mass index (BMI) and player position were provided by the medical staff of the teams. To ensure high *consistency* of data collection, the well educated and motivated physical staff members of the three included teams were invited to a workshop day, where every test was extensively demonstrated and the accompanying test forms to report the results were explained. Afterwards, they were provided with a study manual describing the procedures used to record the data at the defined time frames. Within this study cohort, the timing of the positive PCR tests differed substantially in between individual players. Therefore, no standardized time frames could be reported to indicate when they got infected precisely during the follow-up period and, as such, moving time-windows were used in which the days of quarantine and days till performance tests (proceeding Return to Play clearance after quarantine) were individually registered. The first authors of the study (EW and SD in the authors’ list) were regularly present at test moments and checked all reports monthly and, if necessary, feedback was sent to the teams to check for missing or unclear data.

### Participants

A total of 109 players participated in this study. Goalkeepers and players transferred to another team within the first month after the start of the season were not included, resulting in 84 participants for further analyses. First the staff and then all athletes were informed on the original study design and aim. Every participating player signed an informed consent in accordance with the Declaration of Helsinki. This study was approved by the Ethics Committee of Ghent University (number of approval: B670201940603).

### Patient and public involvement

This study originated as a side project of a larger prospective cohort study on injury prevention in elite male football players [10]. Due to the COVID-19 pandemic, it was decided to develop the current research question in consultation with the medical staff of the participating teams. Therefore, all analyses were performed on a sample of convenience.

### Assessment of SARS-CoV-2 infection

Assessment of SARS-CoV-2 infection was performed by means of a nasal swab-based polymerase chain reaction (PCR) test at least 48 h before each official game. An independent contractor performed a mandatory test on each player at least 48 h before each official game. Because of its high accuracy and reliability, this procedure is considered the gold standard for SARS-CoV-2 testing or for confirming COVID-19 diagnosis [11,12]. If a player tested positive, a second test was performed within 48 h to confirm this positive test result. An overview of the infected players was documented by the team’s physician, including the timing of the positive PCR test(s), the days absent from training and whether the infection caused the COVID-19 disease. In addition, the severity of symptoms (asymptomatic, mild, medium and severe) was registered, based on the classification made by the National Institute of Health (NIH) [13], used as clinical guidelines, since patients with SARS-CoV-2 infection can experience a range of clinical manifestations ranging from an asymptomatic infection to severe illness. Asymptomatic Infection: Individuals who test positive for SARS-CoV-2 but who have no symptoms that are consistent with COVID-19. Mild Illness: Individuals who have any of the various signs and symptoms of COVID-19 (e.g. fever, cough, sore throat, malaise, headache, muscle pain, nausea, vomiting, diarrhoea, loss of taste and smell) but who do not have shortness of breath, dyspnoea, or abnormal chest imaging. Moderate Illness: Individuals who show evidence of lower respiratory disease during clinical assessment or imaging and who have an oxygen saturation (SpO2) ≥94% on room air at sea level. And severe Illness: Individuals who have SpO2 < 94% on room air at sea level, a ratio of arterial partial pressure of oxygen to fraction of inspired oxygen (PaO2/FiO2) <300 mm Hg, respiratory frequency >30 breaths/min, or lung infiltrates >50%. All COVID positive athletes got infected once during the follow-up period.

### Physical performance tests

In a high-intensity intermittent sport such as football, the physical demands are complex, encompassing both high anaerobic and aerobic performance during high-intensity exercise [14]. Therefore, both the athletes’ aerobic and anaerobic performance were monitored in this study, using field tests.

#### Aerobic performance test

**Figure 2.**
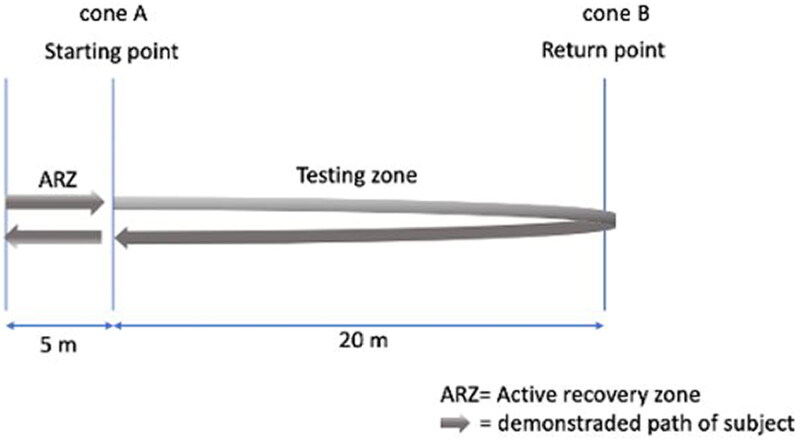
The Yo-Yo intermittent recovery test.

Large significant associations between heart rate (HR) during the submaximal Yo-Yo Intermittent Recovery Test Level 1 (YYIR) and the distance covered in the maximal YYIR test are shown (especially in a trained population), suggesting that the YYIR submaximal test has a predictive validity for the corresponding maximal version [15]. Since elite football clubs often limit the number of occasions they maximally test players due to heavy fixture schedules [16], the submaximal YYIR test was proposed to monitor the players every eight weeks at match day + 2 or 3, after a recuperation training, in order to limit possible interference of previous exercise on these test results. This submaximal YYIR test consist of 2 × 20 m shuttle runs at increasing speed, interspersed with a 10-s period of active recovery (2 × 5m). The aim of this test is to run 720 m in 6 min until the player reaches a speed of 14.5 Km.h^−1^ [17] (Figure 2). The intermittent aerobic performance is evaluated by registering the HR at exactly 3 min (halfway test), 6 min (end of test) and the first and second minute after ending the test. All HR were recorded at 1-s intervals using Polar Team System (Polar Electro Oy, Kempele, Finland). This test has a high reproducibility and sensitivity, allowing detailed analysis of the aerobic performance of athletes in intermittent sports [18]. For consecutive data analysis, these absolute submaximal (3 and 6 min) and recovery (1 and 2 min) HR were normalized to the participant’s maximal HR (%HRmax), to allow valid in-between-subject comparison [15,18–20]. Data on the athletes’ maximal HR was collected during a preseason maximal incremental exercise test (step protocol) on the treadmill, evaluating pulmonary gas exchange and blood lactate concentrations to determine peak oxygen uptake and the aerobic and anaerobic threshold. The following formula was used to calculate %HRmax:

$$\%\mathrm{HRmax}=\frac{\text{submaximal HR}}{\text{maximal HR}}\;\times \;100$$

#### Anaerobic performance tests

##### Strength tests

To assess eccentric knee flexor strength, the NordBord testing system (Vald Performance, Albion, Australia) was used, reported as a reliable system with intraclass correlation coefficient (ICC) values ranging between 0.9 and 0.997 [21,22]. Athletes were positioned in a kneeling position with both ankles fixed and were instructed to gradually lean forward, slowly lowering their trunk position, while keeping neutral hip extension. If the athlete was no longer able to sustain this standardized position, the test was terminated. This was repeated two times and the mean eccentric torque value was calculated. In addition, the GroinBar testing system (Vald Performance, Albion, Australia) was used to examine the isometric strength of the hip adductors and abductors [23]. The GroinBar is stated to be a reliable tool with good to excellent reliability in athletic populations (ICC range from 0.8 to 0.95) [23]. Athletes were in supine position with the knees extended and force pads placed on the lateral and medial malleoli of both legs (bilateral strength measurement). They were then instructed to perform both maximal adduction and abduction efforts for a duration of 3 s, interspersed by a 3 s rest interval. Two repetitions were performed with a rest period of 10 s in between the two repetitions. Mean values for abduction and adduction strength for both legs were calculated.

##### Jump tests

The Countermovement jump (CMJ) was performed using the Optojump (Optojump, Microgate, Bolzano, Italy) to measure maximal vertical jump height (cm) [24]. Athletes started in a standing position and were asked to initiate the CMJ with hip and knee flexion, while extending legs and arms. The mean value of three repetitions was calculated and taken for further analyses. To measure maximal vertical jump height without allowing counter movement related energy storage in the lower limb muscle-tendon system, the Squat jump (SJ) was performed as well [25]. This jump test was also performed using the Optojump system. Athletes started in a position with the knees flexed 90° and a straight back. Then, an explosive vertical jump was performed while extending the legs to land again in the same 90° knee flexion position. Each player performed three maximal jumps of which the mean value was calculated. Both the CMJ and the SJ are reliable and valid tests for the estimation of explosive power of the lower limbs with ICC values between 0.9 and 0.99 [26,27].

##### Sprint tests

The 5- and 20-m sprint tests were performed simultaneously on the field after a standardized warm-up [28]. Players were instructed to run one time as fast as possible through timing gates (Microgate, Bolzano, Italy), which were positioned at the start, at a distance of 5 m and at 20 m. They were positioned after the first timing gates, with their entire body, and started when ready, hereby eliminating reaction time.

### Statistical analysis

Statistical analyses were performed using the SPSS V.27 statistical software (IBM Corp., New York, USA). Descriptive statistics were performed to present player characteristics and SARS-CoV-2 infections overviews. To evaluate the differences in physical performance before and at different measurement points after a SARS-CoV-2 infection, the results of the performance tests in SARS-CoV-2 infected players were compared before and at different measurement points after their SARS-CoV-2 infection using mixed model analyses. The first analysis compared the pre-COVID-19 test data to the first test results after SARS-CoV-2 infection (POST1 COVID-19). Since the timing of the tests was fixed for all athletes, the time till testing after RTS in the infected athletes might vary*.* To have a correct representation of the athlete’s performance capacity after return to sport following COVID-19 infection, athletes in whom the performance tests were performed more than 10 weeks after the positive PCR test were excluded. Pre-post COVID-19 comparison was also performed to compare pre-COVID-19 test data with the second measurement after infection (POST2 COVID-19) to verify to what extent physical performance potentially recovered proceeding infection. Mixed models analyses both within the isolated groups of SARS-CoV-2 infected players as in the group of non-infected players were executed with participants and teams as random factors and moment of measurements (pre-COVID-19, POST1 COVID-19 and POST2 COVID-19) as fixed predictor. In addition, mixed models analyses were also performed to examine the difference of the physical performance variables between the players who tested positive on the PCR test and their COVID-19 negative teammates at the same time frames. These analyses were executed in all athletes with participants and teams as random factors and COVID-19 as fixed predictor. Post hoc analyses were performed using Bonferroni corrections [29]. The effect of possible covariates as age, BMI, severity of symptoms, time till testing after RTS (after infection) and playing position was investigated. The residuals of the linear mixed model were checked for normal distribution and homoscedasticity. The level of significance was set at α = 0.05.

## Results

In total, 22 of the 84 included athletes got infected with SARS-CoV-2 during the follow-up period. The majority of the infected players (82%) developed symptoms and the overall average quarantine duration was 12.1 ± 6.1 days. Subject demographics and SARS-CoV-2 infections overview per team can be found in Table 1. BMI, age, severity of symptoms, time till testing after RTS (after infection) and playing position did not have an effect on the outcome parameter and are therefore not included as covariates in the analyses.

**Table 1.** Participant demographics, injury and SARS-CoV-2 infections overview per team.

Subjects characteristics			
	Team A (*n* = 32)	Team B (*n* = 30)	Team C (*n* = 22)
	Mean ± SD	Mean ± SD	Mean ± SD
Age (y)	23.72 ± 3.85	24.97 ± 4.34	25.50 ± 5.46
Height (m)	1.82 ± 0.68	1.83 ± 0.77	1.83 ± 0.59
Weight (kg)	77.85 ± 0.85	78.61 ± 0.83	77.90 ± 0.76
BMI (kg/m²)	23.40 ± 1.36	23.58 ± 1.53	23.31 ± 1.50
Games/ week (n)	1.70 ± 0.55	1.64 ± 0.56	1.23 ± 0.43
COVID-19 infections			
	Count (%)	Count (%)	Count (%)
Number of infected players (n)	10	10	2
Symptomatic			
Yes	8 (80)	8 (80)	2 (100)
No	2 (20)	2 (20)	0 (0)
Severity of the symptoms			
Mild	1 (10)	5 (50)	1 (50)
Moderate	5 (50)	3 (30)	1 (50)
Severe	2 (20)	.	.
	Mean ± SD	Mean ± SD	Mean ± SD
Quarantine duration (days)	15.03 ± 7.06	9.00 ± 3.52	15.00 ± 0.0

*Notes:* Team A, B and C represent the three participating teams, pseudonymized. BMI: body mass index; SARS-CoV-2: Severe Acute Respiratory Syndrome Coronavirus 2; SD: standard deviation.

At the first testing after a SARS-CoV-2 infection (POST1), significantly higher %HRmax were registered (compared to pre-infection) at 6 min during (*p* = .006) and at 1 and 2 min after the YYIR test (*p* = .045 and *p* = .013, respectively) (Table 2). The POST1%HRmax values measured at 3 min during the test showed non-significantly higher values (*p* = .083) (Table 2). POST1 YYIR tests were performed averagely 52.0 ± 11.2 days after athletes tested positive on the PCR tests. Comparing the pre-infection normalized YYIR HR values with second post-infection YYIR values (POST2) revealed no statistical differences (*p* values ranging from .205 to .833) (Table 2). POST2 YYIR tests were performed averagely 127.6 ± 33.1 days after positive PCR testing within this group of formerly infected players.

**Table 2.** The YYIR %HRmax in SARS-CoV-2 infected athletes over time.

					95% Confidence interval	
		b	SE b	*p* Value	Lower bound	Upper bound
**%HRmax at 3 min**	Intercept	79.70	1.70	<.001	76.18	83.20
Halfway test	**Pre COVID = ref category**					
	Post 1 COVID	3.91	2.11	.083	−0.57	8.40
	Post 2 COVID	−3.74	2.84	.205	−9.72	2.24
**%HRmax at 6 min** End of test	Intercept	84.91	1.19	<.001	82.43	87.39
	**Pre COVID = ref category**					
	Post 1 COVID	4.47	1.87	**.006**	1.53	7.41
	Post 2 COVID	−1.95	1.38	.311	−5.91	2.00
**%HRmax 1 min after test**	Intercept	63.50	2.42	<.001	58.48	68.51
	**Pre COVID = ref category**					
	Post 1 COVID	5.80	2.68	**.045**	0.14	11.46
	Post 2 COVID	0.78	3.64	.833	−6.87	8.42
**%HRmax 2 min after test**	Intercept	55.29	2.08	<.001	50.89	59.69
	**Pre COVID = ref category**					
	Post 1 COVID	4.61	1.65	**.013**	1.10	8.12
	Post 2 COVID	2.16	2.26	.354	−2.64	6.96

*Notes: p* Values were obtained by means of mixed models analyses in the SARS-CoV-2 infected players and p-values <0.05 are in bold. %HRmax: percentage of maximal heart rate; *b*: *%HRmax supplemented by the differences in %HRmax over time*; Pre COVID: before the SARS-CoV-2 infection; Post 1 COVID: first post-infection test moment; Post 2 COVID: second post-infection test moment; Ref category: reference category; *SARS-CoV-2: Severe Acute Respiratory Syndrome Coronavirus 2; SE b: the accompanying standard error; YYIR: Yo-Yo Intermittent Recovery test*.

This finding is in contrast to the significant decrease in %HRmax found in the non-infected population at the same time interval (at 3 min during the test; *p* = .032 with a decrease of 3%, at 6 min during the test; *p* = .002 with a decrease of 2%, at 1 min after the test; *p* = .003 with a decrease of 3% and for the measuring point 2 min after the test; *p* = .004 with a decrease of 5%) (Table 3).

**Table 3.** The YYIR %HRmax in non-infected athletes over time.

					95% Confidence interval	
		b	SE b	*p* Value	Lower bound	Upper bound
**%HRmax at 3 min**	Intercept	77.64	0.89	<.001	75.84	79.44
Halfway test	**Pre = ref category**					
	Post	−2.54	1.11	**.032**	−4.85	−0.24
**%HRmax at 6 min** End of test	Intercept	85.20	0.73	<.001	83.73	86.67
	**Pre = ref category**					
	Post	−1.76	0.53	**.002**	1.53	7.41
**%HRmax 1 min after test**	Intercept	64.59	1.26	<.001	62.06	67.12
	**Pre = ref category**					
	Post	−3.47	1.08	**.003**	−5.65	−1.28
**%HRmax 2 min after test**	Intercept	53.67	1.68	<.001	50.25	57.09
	**Pre = ref category**					
	Post	−5.29	1.66	**.004**	−8.75	−1.84

*Notes: p* Values were obtained by means of mixed models analyses in the non-infected players and *p*-values <0.05 are in bold. %HRmax: percentage of maximal heart rate; *b: %HRmax supplemented by the differences in %HRmax over time*; Pre: before the SARS-CoV-2 infection of the infected group; Pos: first test moment after the SARS-CoV-2 infection of the infected group; Ref category: reference category; *SARS-CoV-2: Severe Acute Respiratory Syndrome Coronavirus 2*; *SE b is the accompanying standard error*.

**Figure 3.**
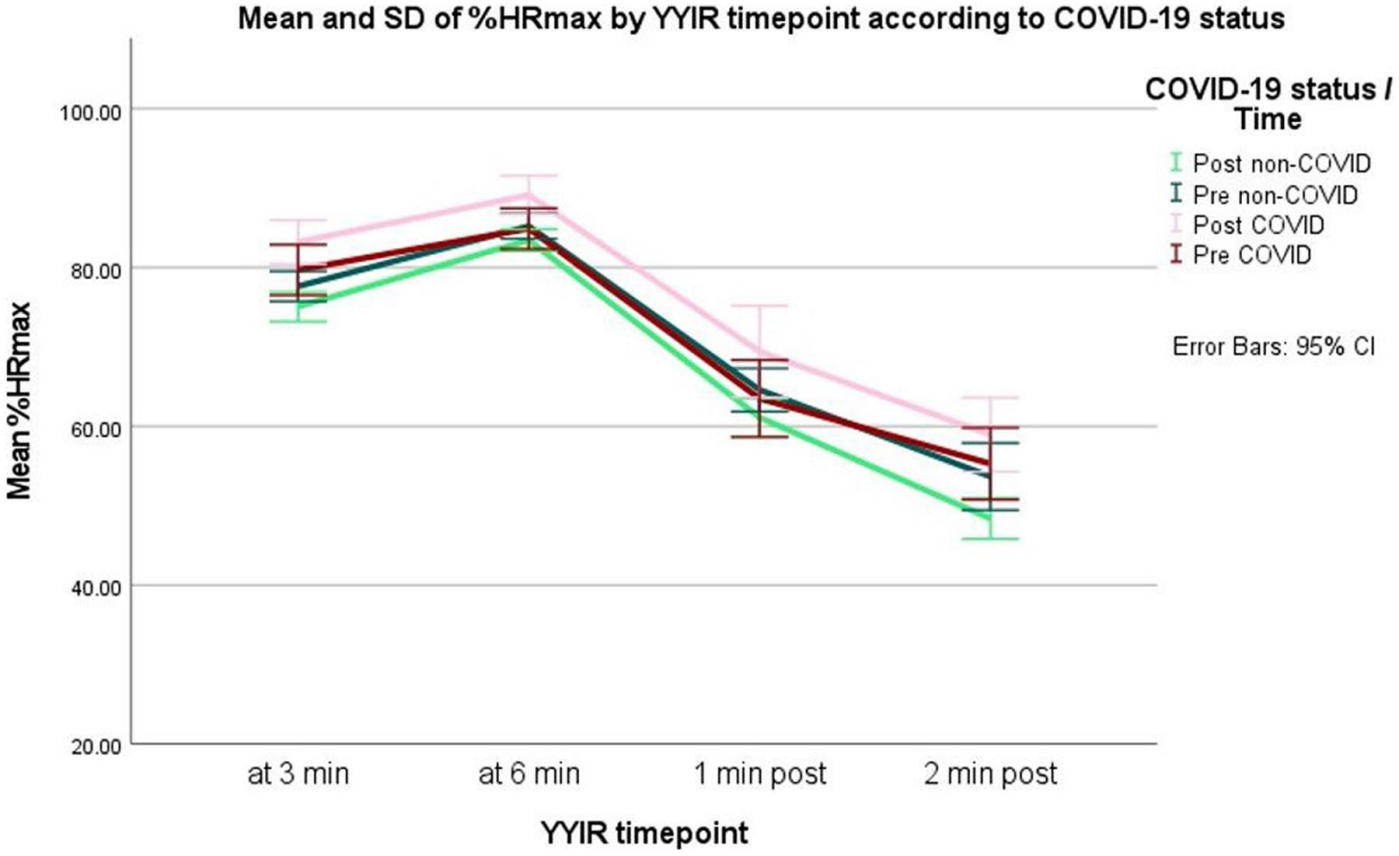
Mean and SD of %HRmax by YYIR timepoint according to COVID-19 status.

In addition, the %HRmax values during the YYIR test were significantly higher (ranging from 6 to 11%) in formerly COVID-19 positive players, compared to their COVID-19 negative teammates, and this at all measuring points (3 and 6 min during; *p* < .001, and at the measuring points 1 min after; *p* = .002 and 2 min after the test; *p* < .001) (Table 4). These significant differences between the formerly infected players and the non-infected players were only established at the POST1 YYIR testing session (52.0 ± 11.2 days after positive PCR testing). No significant differences in %HRmax values of the YYIR test before the SARS-CoV-2 infection (*p* values ranging from .417 to .842), and at POST2 YYIR testing between infected and non-infected players were observed (127.6 ± 33.1 days after positive PCR testing) (*p* values ranging from .650 to .965) (Table 4 and Figure 3).

**Table 4.** Results of the YYIR %HRmax between formerly COVID-19 positive and non-infected players.

					95% Confidence Interval	
		b	SE b	*p* Value	Lower Bound	Upper Bound
**%HRmax at 3 min**	Intercept	83.21	1.24	<.001	80.86	85.74
Halfway test	**COVID infected = ref category**					
	Non-infected athletes	−8.14	1.53	**<.001**	−11.27	−5.02
**%HRmax at 6 min** End of test	Intercept	89.12	1.22	<.001	86.66	91.58
	**COVID infected = ref category**					
	Non-infected athletes	−5.68	1.39	**<.001**	−8.48	−2.88
**%HRmax 1 min after test**	Intercept	69.41	2.26	<.001	64.86	73.96
	**COVID infected = ref category**					
	Non-infected athletes	−8.30	2.58	**.002**	−13.48	−3.11
**%HRmax 2 min after test**	Intercept	58.93	1.86	<.001	55.09	62.78
	**COVID infected = ref category**					
	Non-infected athletes	−10.56	2.28	**<.001**	−15.21	−5.90

*Notes: p* Values were obtained by means of mixed models analyses in all athletes and *p*-values <0.05 are in bold. %HRmax: percentage of maximal heart rate; *b: %HRmax supplemented by the differences in %HRmax between groups;* Ref category: reference category*; SE b is the accompanying standard error*.

In this elite population, no significant differences before and after SARS-CoV-2 infections were found for the jump tests (*p* = .102 for the CMJ and *p* = .154 for the SJ), nor for the strength tests (*p* = .879 for the hamstring strength, *p* = .383 for the adductor strength and *p* = .103 for the abductor strength) or the sprint tests (*p* = .124 for the 5 m and *p* = .449 for the 20 m sprint). Post-infection jump, muscular strength and sprint tests were performed averagely 53.9 ± 9.6, 48.2 ± 6.3 and 55.4 ± 8.3 days after positive PCR testing, respectively.

## Discussion

The results of this study showed that the aerobic performance is lower after a SARS-CoV-2 infection in elite football players*.* This increase in %HRmax during the YYIR test was only seen at the first testing after the SARS-CoV-2 infection (averagely 52.0 ± 11.2 days after positive PCR testing) and resolved at the second testing after infection (averagely 127.6 ± 33.1 days after positive PCR testing). These findings are in contrast to the slight improvement in YYIR performances found in the non-infected players, indicated by a 2–5% decrease in %HRmax, which is in accordance with previous literature in elite soccer players [30]. Important to note is that a difference of 2.5% in %HRmax is clinically significant in elite athletes [21], indicating that our findings are clinically relevant.

### Effect of SARS-CoV-2 infection on aerobic performance

The results of this study showed significantly higher %HRmax at 6 min during and at 1 and 2 min after the submaximal YYIR test. The non-significant higher %HRmax at 3 min during the YYIR test could be explained by the duration since 3 min is not enough to sufficiently speed-up the cardiovascular system [16,18]*.* In addition, formerly infected players showed significantly higher %HRmax (ranging from 6 to 11%) during and after the YYIR test compared to the non-infected players at the first testing after infection. Several possible explanations for this have been previously reported.

It has been previously stated that acute infective illness can cause a reduction in exercise performance through different mechanisms including a decrease in endurance capacity due to alterations in muscle enzyme activity and metabolic function [31]. More specifically, SARS-CoV-2 infection has been shown to induce capillary flow disturbances, which are shown to shorten blood transit times through the remaining, patent capillaries, thereby limiting oxygen uptake [32]. So, these capillary disturbances are expected to reduce the endurance capacity of elite players [32,33]. Also, a decrease in maximal aerobic performance after a SARS-CoV-2 infection was found in a 2020 study on military recruits [33]. The results of this study are also confirmed in the Moulson et al. study [9] where spirometry revealed that approximately one-third of formerly infected athletes had evidence of airflow obstruction and many had a reduced breathing reserve at peak exercise still three months after diagnosis.

A second possible explanation for the results might be fatigue since, following the acute phase of COVID-19, there are now consistent reports that COVID-19 positive athletes may present persistent and residual symptoms for weeks to months after quarantine, including cough, tachycardia and (extreme) fatigue [34]. More so, it is well established that fatigue negatively affects aerobic performance of athletes [35]. The fact that these decrements in intermittent aerobic performance (i.e. YYIR) seem to vanish with time (POST2 COVID-19 YYIR did not reveal any residual increments in %HRmax during or after YYIR testing), is however reassuring.

In addition to the pathophysiological effects of SARS-CoV-2, the detraining effects of the obliged quarantine period after positive COVID-19 testing might also impact this decrement in aerobic performance [36]. Also, a (too) fast RTP (averagely 12.1 ± 6.1 days after positive testing) at previous level of training and competition after infection should be considered [28]. The results of our study showed a disbalance in external/internal load applied. As the external load during testing (physical, e.g*.* distance and speed applied during the submaximal YYIR test) did not differ, the internal load (physiological, HR%) significantly differed after infection. Therefore, we advocate for a gradual built- up (specifically adapted to the athletes’ load bearing capacities at that time) since it seems possible that the athletes were not sufficiently recovered and physically capable of rejoining their team at former and generic levels of training intensity. Since training effects are prone to the athletes’ starting status [37]*,* the established decrements in YYIR performance might therefore partially be the consequence of an insufficient recovery and a rapid RTP [38]. In their practical guide concerning cardiorespiratory considerations prior to safe RTP in athletes, Wilson et al. [34] focus on the importance of thorough clinical and cardiorespiratory examination and gradual progression of exposure to physical activity in general and football-specific activities in particular, in function of safe RTP. According to a 2020 study of Elliot et al. [7], a graduated RTP protocol implies the inclusion of at least 7 days of rest after disappearance of symptoms or after 10 days in case of early symptom resolution. This implies a minimal resting period of 17 days, which should then be followed by a six-stage gradual sports exposure trajectory, implying re-uptake of light physical activity and step-wise reintegration in regular training activities by systematically increasing [1] frequency [2], duration and [3] intensity of training sessions and ultimately re-participation at former level of training and competition [7].

### Effect of SARS-CoV-2 infection on the anaerobic performance tests

Finally, in this study, no remaining deficits were found for the anaerobic performance tests. This is similar to previous research that also found that anaerobic performance did not seem to be affected by COVID-19 [33]. A possible explanation for this finding might be the timing of these measurements since it is possible that – in contrast to the aerobic performance decrements – potential present decrements in *jump, muscular strength and sprint* tests after infection were already resolved at the time of testing [33]. This is assumed since the mandatory quarantine period for the infected athletes may induce a certain degree of short-term muscle impairment, particularly since at this time, the effect of early physical activity following SARS-CoV-2 infection was unclear, the team’s M.D. urged the players not to engage in physical activity during their obligated quarantine period, potentially resulting in partial or complete loss of exercise-induced morphological and physiological adaptations [34]. A previous study of de Boer et al. [39] determined that during a suspension period, an average lower limb strength loss of 1.06% a day was found over the first two weeks and a 0.68% strength loss a day the following nine days [35]. It should be noted that this concerned a unilateral suspension (while detraining refers to an insufficient training stimulus) in healthy men. Nonetheless, previous research in the same cohort demonstrated that these changes can be reversed with active recovery, with muscle function fully restored three weeks post disuse [40]. In our study, the athletes performed these jump, muscular strength and sprint tests on average 53.9 ± 9.6, 48.2 ± 6.3 and 55.4 ± 8.3 days after a positive PCR test, respectively, indicating that they were already training for more than five weeks – after a quarantine duration of averagely 12.1 ± 6.1 days – at the time of testing. Moreover, previous research in a (highly) trained athletic population even showed that short term detraining (<4 weeks) in trained athletes could lead to maintenance for anaerobic performance [2], potentially also explaining why anaerobic performance is not affected in this study*.* Another possible explanation might be that the most commonly reported persistent (LC19) symptoms are well established to affect athlete’s aerobic performance (as previously described) [35], but seems to spare anaerobic performance (such as jump, muscular strength and sprint tests) [41].

### Clinical implications

Previous research showed that moderate physical activity promotes a healthy immunological response to infection and possibly suppresses autoimmune activity in the absence of infection [6]. However, caution is needed considering the volume and/or intensity of the physical activity since high-intensity and/or prolonged exercise leads to immunosuppression [6,42,43]. A 2021 review regarding the impact of exercise on post COVID-19 syndrome stated that exhaustive and excessive exercise training may impair mitochondrial function, resulting in a dysregulated systemic inflammatory response with potentially adverse health consequences [44]. Since (elite) athletes feel pressured to return to training and competition as fast as possible, they often exceed the physical activity recommendations after SARS-CoV-2 infection for physical activity to be health-promoting [42,45]. Even more, their strenuous training program might be potentially detrimental since heavy exercise workloads after a SARS-CoV-2 infection are stated to result in an extensive downturn in the function of innate immune system cells including macrophages, neutrophils and natural killer cells [46]. Based on this, it seems possible that this potentially detrimental effect of strenuous exercise after infection might explain why acute COVID*-*19 symptoms are often mild or even absent in an athletic population but, strikingly, evolve to persisting symptoms after SARS-CoV-2 infection in a considerable proportion of athletes [3,46].

The results of this study showed that aerobic performance in elite athletes remained impaired for approximately 50 days after infection, indicating that caution might be needed with strenuous and prolonged exercise during this time especially since this could potentially undermine the immune system. Therefore, the authors would advise clinicians and trainers to organize RTP after SARS-CoV-2 infection with gradual built-up, allowing athletes to participate in (submaximal) training sessions between approximately 17 and 50 days after positive testing, but to remain cautious with high-intensity exposure during this period. Regular YYIR testing is recommended to monitor aerobic performance and to evaluate until pre-infection level is reached, which seems beneficial to justify clearance for maximal exposure.

### Methodological considerations

Although this was the first prospective study assessing the impact of COVID-19 on intermittent aerobic performance and anaerobic performance in a cohort of elite football players, it is not without limitations. Firstly, the direct and isolated effect of COVID-19 on performance should be investigated in future research since this study design did not allow us to make direct causal inference. Secondly, this study is based on a rather low number of SARS-CoV-2 positive cases, and therefore caution is needed when generalizing these results. We would recommend future research to examine these findings in a study with a larger sample size and investigate this in different sports as well. Thirdly, subjective complaints are hard to report in a competitive athletic population, nonetheless, this would have been an added value to the study. Next, monitoring of physical performance was conducted by means of user-friendly submaximal field-tests, instead of more valid and reliable maximal and/or laboratory tests in highly standardized and controlled settings. The authors chose to do so because this is the most common practical approach practiced by physical, technical and medical staffs at elite level, and it facilitates and accelerates data collection and interpretation. The implementation of perceived exertion rating or similar scoring systems could also provide valuable insights in the load bearing capacities of the athletes after RTP.

## Conclusion

The findings of the current study demonstrated that the aerobic performance seems compromised for approximately seven weeks after sports resumption after SARS-CoV-2 infection. At this time, anaerobic performance seemed to be spared. Because of the potential detrimental effects of strenuous exercise after infection on the immune system, caution might be needed until aerobic performance is fully restored. Regular aerobic capacity testing (e.g. YYIR) is recommended to monitor aerobic performance, preferably at regular time intervals throughout the season so aerobic capacity after SARS-CoV-2 infection can be compared to pre-infection aerobic capacity within infected athletes, in addition to the comparison with non-infected counterparts*.*

## Supplementary Material

Supplemental Material

## Data Availability

Data are available upon reasonable request. Requests for data sharing from appropriate researchers and entities will be considered on a case-by-case basis. Interested parties should contact Dr Wezenbeek (evi.wezenbeek@ugent.be).
