# Supplementary material for: Impact of (long) COVID on athletes’ performance: a prospective study in elite football players
Source: Ann Med. 2023 Apr 26;55(1):2198776. doi: 10.1080/07853890.2023.2198776 (PMC10134946; doi:10.1080/07853890.2023.2198776)
Supplement: Supplemental Material [file IANN_A_2198776_SM0915.pdf]

Afz: Commissie voor Medische Ethiek

COMMISSIE VOOR MEDISCHE ETHIEK

Voorzitter:
Prof. Dr. D. Matthys
Secretaris:
Prof. Dr. J. Decruyenaere

REVAKI
Kliniekgebouw 3 - 6 de Verdieping
Prof. dr. Erik WITVROUW
ALHIER

**CONTACT**	**TELEFOON**	**FAX**	**E-MAIL**
Secretariaat	+32 (0)9 332 56 13<br>+32 (0)9 332 59 25	+32 (0)9 332 49 62	ethisch.comite@ugent.be
**UW KENMERK**	**ONS KENMERK**	**DATUM**	**KOPIE**
	2019/0886	13-aug-19	Zie "CC"

BETREFT

Advies voor monocentrische studie met als titel:
Jupiler Pro League Injury Prevention Project

Belgisch Registratienummer: B670201940603

Fase (Phase): NVT/NA

* Begeleidende brief dd. 20/05/2019
* Diverse
  - Term sheet UGent - Football clubs injury prevention study
* Antwoord onderzoekers
  dd 12/08/2019 op opmerkingen EC dd 01/07/2019
* Adviesaanvraagformulier dd. 08/08/2019
  (Volledig ontvangen dd 12/08/2019) Versie 2
* (patiënten)- informatie en toestemmingsformulier dd. 08/08/2019
  Versie 2 NL, ENG

Advies werd gevraagd door:
Prof. dr. E. WITVROUW ; Hoofdonderzoeker

BOVENVERMELDE DOCUMENTEN WERDEN DOOR HET ETHISCH COMITÉ BEOORDEELD.
ER WERD EEN POSITIEF ADVIES GEGEVEN OVER DIT PROTOCOL OP 13/08/2019. INDIEN DE STUDIE NIET WORDT OPGESTART VOOR 12/08/2020, VERVALT HET ADVIES EN MOET HET PROJECT TERUG INGEDIEND WORDEN.
Vooraleer het onderzoek te starten dient contact te worden genomen met Bimetra Clinics (09/332 05 00).

THE ABOVE MENTIONED DOCUMENTS HAVE BEEN REVIEWED BY THE ETHICS COMMITTEE.
A POSITIVE ADVICE WAS GIVEN FOR THIS PROTOCOL ON 13/08/2019. IN CASE THIS STUDY IS NOT STARTED BY 12/08/2020, THIS ADVICE
WILL BE NO LONGER VALID AND THE PROJECT MUST BE RESUBMITTED.
Before initiating the study, please contact Bimetra Clinics (09/332 05 00).

DIT ADVIES WORDT OPGENOMEN IN HET VERSLAG VAN DE VERGADERING VAN HET ETHISCH COMITE VAN 20/08/2019
THIS ADVICE WILL APPEAR IN THE PROCEEDINGS OF THE MEETING OF THE ETHICS COMMITTEE OF 20/08/2019

CONTACT	TELEFOON	FAX	E-MAIL
Secretariaat	+32 (0)9 332 56 13<br>+32 (0)9 332 59 25	+32 (0)9 332 49 62	ethisch.comite@ugent.be
**UW KENMERK**	**ONS KENMERK**	**DATUM**	**KOPIE**
	2019/0886	13-aug-19	Zie "CC"

- *Het Ethisch Comité werkt volgens 'ICH Good Clinical Practice' - regels*
- *Het Ethisch Comité beklemtoont dat een gunstig advies niet betekent dat het Comité de verantwoordelijkheid voor het onderzoek op zich neemt. Bovendien dient U er over te waken dat Uw mening als betrokken onderzoeker wordt weergegeven in publicaties, rapporten voor de overheid enz., die het resultaat zijn van dit onderzoek.*
- *In het kader van 'Good Clinical Practice' moet de mogelijkheid bestaan dat het farmaceutisch bedrijf en de autoriteiten inzage krijgen van de originele data. In dit verband dienen de onderzoekers erover te waken dat dit gebeurt zonder schending van de privacy van de proefpersonen.*

- *Het Ethisch Comité benadrukt dat het de promotor is die garant dient te staan voor de conformiteit van de anderstalige informatie- en toestemmingsformulieren met de nederlandstalige documenten.*
- *Geen enkele onderzoeker betrokken bij deze studie is lid van het Ethisch Comité.*
- *Alle leden van het Ethisch Comité hebben dit project beoordeeld. (De ledenlijst is bijgevoegd)*

- *The Ethics Committee is organized and operates according to the 'ICH Good Clinical Practice' rules.*
- *The Ethics Committee stresses that approval of a study does not mean that the Committee accepts responsibility for it. Moreover, please keep in mind that your opinion as investigator is presented in the publications, reports to the government, etc., that are a result of this research.*

- *In the framework of 'Good Clinical Practice', the pharmaceutical company and the authorities have the right to inspect the original data. The investigators have to assure that the privacy of the subjects is respected.*
- *The Ethics Committee stresses that it is the responsibility of the promotor to guarantee the conformity of the non-dutch informed consent forms with the dutch documents.*
- *None of the investigators involved in this study is a member of the Ethics Committee.*
- *All members of the Ethics Committee have reviewed this project. (The list of the members is enclosed)*

Namens het Ethisch Comité / On behalf of the Ethics Committee

Prof. dr. D. MATTHYS
Voorzitter / Chairman

CC: De heer T. VERSCHOORE - UZ Gent - Bimetra Clinics
FAGG - Research & Development; Victor Hortaplein 40, postbus 40 1060 Brussel
